# Prevalence of cognitive impairment in Chinese older inpatients and its relationship with 1-year adverse health outcomes: a multi-center cohort study

**DOI:** 10.1186/s12877-021-02556-5

**Published:** 2021-10-25

**Authors:** Li Yuan, Xiaoming Zhang, Na Guo, Zhen Li, Dongmei Lv, Hui Wang, Jingfen Jin, Xianxiu Wen, Shengxiu Zhao, Tao Xu, Jing Jiao, Xinjuan Wu

**Affiliations:** 1 Department of Epidemiology and Statistics, Institute of Basic Medical Sciences, Chinese Academy of Medical Sciences & School of Basic Medicine, Peking Union Medical College, 5 Dongdan Santiao, Beijing, 100005 China; 2 Department of Nursing, Chinese Academy of Medical Sciences - Peking Union Medical College, Peking Union Medical College Hospital, Beijing, 100730 China; 3 Department of Nursing, The Second Affiliated Hospital of Harbin Medical University, Harbin, China; 4 Department of Nursing, Tongji Hospital, Tongji Medical College, Huazhong University of Science and Technology, Wuhan, China; 5 Department of Nursing, The Second Affiliated Hospital Zhejiang University School of Medicine, Hangzhou, China; 6 Department of Nursing, Sichuan Provincial People’s Hospital, Chengdu, China; 7 Department of Nursing, Qinghai Provincial People’s Hospital, Xining, China

**Keywords:** Cognitive impairment, Older inpatients, Adverse outcomes, Mortality, Frailty, HRQoL

## Abstract

**Background:**

Previous studies on the relationship between cognitive impairment and adverse outcomes among geriatric inpatients are not representative of older inpatients in China because of insufficient sample sizes or single-center study designs. The purpose of our study was to examine the prevalence of cognitive impairment and the relationship between cognitive impairment and 1-year adverse health outcomes in older inpatients.

**Methods:**

This study was a large-scale multi-center cohort study conducted from October 2018 to February 2020. Six tertiary hospitals across China were selected using a two-stage cluster sampling method, and eligible older inpatients were selected for the baseline survey and follow-up. The Mini Cognitive Scale and the FRAIL scale were used to screen for cognitive impairment and frailty, respectively. The EuroQol-5 Dimension-5 Level questionnaire was used to assess health-related quality of life (HRQoL). We used a generalized estimating model to evaluate the relationship between cognitive impairment and adverse outcomes.

**Results:**

The study included 5008 men (58.02%) and 3623 women (41.98%), and 70.64% were aged 65–75 years, and 26.27% were aged 75–85 years. Cognitive impairment was observed in 1756 patients (20.35%). There were significant differences between participants with cognitive impairment and those with normal cognitive function for age, gender, surgery status, frailty, depression, handgrip strength and so on. After adjusting for multiple covariates, compared with patients with normal cognitive function, the odds ratio for 1-year mortality was 1.216 (95% confidence interval [CI]: 1.076–1.375) and for 1-year incidence of frailty was 1.195 (95% CI: 1.037–1.376) in patients with cognitive impairment. Similarly, the regression coefficient of 1-year HRQoL was − 0.013 (95% CI: − 0.024−− 0.002). In the stratified analysis, risk of adverse outcome within 1 year was higher in older patients with cognitive impairment aged over 75 years than those aged 65–74 years.

**Conclusions:**

We revealed that cognitive impairment was highly correlated with occurrence of 1-year adverse health outcomes (death, frailty, and decreased HRQoL) in older inpatients, which provides a basis for formulating effective intervention measures.

**Trial registration:**

Chinese Clinical Trial Registry, ChiCTR1800017682, registered 09 August 2018.

## Background

The number of elderly people aged over 60 years in China was 249.49 million in 2018 [1], and prevalence of mild cognitive impairment (MCI) in those aged 65 years and above was 20.8% [2]. Cognitive impairment has become an important public health problem in China. The syndrome is characterized by decline in learning, memory, language, attention, social cognition, and other abilities [3]. The cognitive function of the patients is lower than the general level of their peers, but it has no significant effect on activities of daily living (ADL). It is a transitional stage between normal cognition and dementia, and the annual rate of progression to Alzheimer’s disease is 18% [4]. Individuals will be diagnosed with dementia when cognitive decline affects ADL, such as eating and dressing [5]. Risk factors of cognitive impairment include advanced age, obesity, heart disease, diabetes, depression, and physical frailty [3]. The reduction in cognitive ability and dependence on nursing are long-term issues for patients and caregivers, which impose a significant burden on families and society [6].

Studies have shown that cognitive impairment is significantly associated with risk of adverse health outcomes [7, 8]. Adverse health outcomes include mortality [9], rehospitalization, frailty, and decreased health-related quality of life (HRQoL). The mechanism of adverse health outcomes due to cognitive impairment is multifactorial. Some researchers have suggested that cognitive impairment is a form of pathological aging of the brain [10], whereas others have indicated that nerve injury reduces the ability to gradually recover from previous diseases [7]. In addition, age has been shown to be an important risk factor for geriatric diseases. As people age, cognitive function naturally declines, and the prevalence of disease multiplies [5]. Gender, depression, and surgical history have also been shown to be related to cognitive decline [11].

Compared with the elderly in the community, syndromes such as depression and dementia are more common among elderly patients in general hospitals [12]. Moreover, in elderly hospitalized patients, cognitive impairment will increase the risk of another senile syndrome during hospitalization and is associated with poor prognosis [7, 12]. However, a survey in Greece found that medical workers seriously underestimated the cognitive decline of elderly inpatients [13]. According to the research of Constantine et al. [14], after an early screening and intervention program, the length of stay and costs for people with dementia have decreased.

Previous studies on the association between cognitive function and prognosis focused on the elderly in the community and were not representative of older inpatients nationwide because of insufficient sample sizes or single-center study designs. Our study was a large-scale multi-center cohort study using a representative elderly hospitalized inpatients sample. In addition, adverse outcomes such as death, frailty, and decreased HRQoL were considered in this study, while most studies focused on only one of these outcomes. Moreover, we used generalized estimating equation (GEE) models to determine the relationship between cognitive impairment and adverse health outcomes in older inpatients. Understanding such associations will help guide the development of therapeutic regimens and implementation of preventive interventions, which will ultimately improve the life expectancy of the elderly population.

## Methods

### Sample and participants

The sample population of this study was older inpatients in tertiary hospitals. The study was a large-scale multi-center cohort study conducted from October 2018 to February 2020 to investigate the psychological and physical conditions of elderly hospitalized patients in China. Baseline data, such as demographic indicators and physiological and psychological conditions, were collected from face-to-face questionnaire interviews, physical examinations, clinical records and clinical assessments. To ensure representativeness of the older inpatients, we adopted a two-stage cluster sampling method. In the first stage, six provinces or municipalities, which were Beijing (North China), Heilongjiang Province (Northeast), Qinghai Province (Northwest), Zhejiang Province (East China), Hubei Province (South China), and Sichuan Province (Southwest), were selected by simple random sampling. In the second stage, simple random sampling was used to select one hospital from qualifying tertiary hospitals within each province or municipality. Participants were recruited from surgery, intensive care, neurology, orthopedic, or internal medicine departments from the six hospitals that met the criteria and totaled 10,000 patients. Inclusion criteria were: ≥ 65 years old; voluntary participation in the study and able to sign informed consent; no persistent disorder of consciousness or communication; able to communicate effectively and caregivers able to provide accurate information. Patients were followed up by telephone at 3 months, 6 months and 1 year after the start of the study. To ensure reliability of data, we compiled survey, operation, and training manuals, selected one to two nurses from each department for follow-up assessments, and comprehensively trained 589 investigators. This study was approved by the ethics committee of Peking Union Medical College Hospital (S-K540).

### Measurement instruments

The Mini Cognitive Scale (Mini-Cog) is a cognitive impairment screening tool for evaluating cognitive function [15]. Compared with the Mini-Mental State Examination (MMSE), the Mini-Cog is faster, simpler, and easier to administer as a screening tool for cognitive impairment [16], and is accepted by both inpatients and outpatients. The Mini-Cog is less affected by language and education [17], and spend less time, which need only 2-4 min to complete [18]. It includes recalling three unrelated words and a clock-drawing test. The patient received 1 point for recalling a word. A patient who scores 0 or scores 1-2 points but performs poorly on the clock drawing test is considered to have cognitive impairment. If a patient scores 3 points or scores 1-2 points and draws a correct clock, they are considered to have no cognitive impairment. In a cross-sectional study of the elderly in eastern China [19], the Mini-Cog was verified to have excellent screening characteristics (area under the curve = 86.52%, sensitivity = 87.61%, specificity = 85.30%).

The FRAIL scale is a screening tool for frailty, which consists of five questions [20]. Each item is scored 0 or 1 point. A total score in five items of 0 indicates robustness, 1-2 indicates pre-frailty, and 3-5 indicates frailty. In a cross-sectional study of the elderly in Chinese communities, the FRAIL scale was verified to have good reliability and validity [21]. It showed good test-retest reliability (intraclass correlation coefficient = 0.708), acceptable convergent validity, satisfactory diagnostic accuracy (area under the curve = 0.91), fair agreement with the Fried frailty phenotype (kappa = 0.274, *P* < 0.001), and good known-group divergent validity (more frail individuals were recognized by the Chinese FRAIL scale among older and female participants than their counterparts).

The EuroQol-5 Dimension-5 Level questionnaire (EQ5D-5 L) measures HRQoL of elderly hospitalized patients [22]. The scale has five dimensions: mobility, self-care, daily activities, pain/discomfort, and anxiety/depression. Each dimension has five levels: no difficulty, a little difficulty, moderate difficulty, serious difficulty, and very serious difficulty. The full score of the scale is 100 points, and higher scores indicate better health. EQ5D is widely used to test HRQoL [23]. It is a general scale, which does not distinguish between MCI patients and participants with normal cognitive function. It is short, easy to use and has good feasibility, acceptability and reliability [24]. In a cross-sectional study of an urban general population in China [25], it showed moderate level of test–retest reliability. Kappa values were from 0.35 to 1.0. The ICCs of test–retest reliability were 0.53 and 0.87 for the EQ-5D index score and for the EQ VAS score respectively. It also demonstrated acceptable construct validity. The Pearson’s correlation coefficients between the EQ-5D and the SF-36 were stronger between comparable dimensions than those between less comparable dimensions.

### Definition of covariates

Factors that may have been associated with cognitive impairment included age (65–74 years, 75–84 years, ≥ 85 years), gender (female or male), ethnicity (Han or others), marital status (married, divorced, or widowed), educational level (illiterate, primary school, middle school, or university), body mass index (BMI; underweight, normal, overweight, or obese), smoking status (current smoker, former smoker, or non-smoker), drinking status (current drinker, former drinker, non-drinker), surgery (yes or no), bedridden for ≥4 weeks (yes or no), falls in the past year (yes or no), handgrip strength (low or normal), frailty (frail, pre-frail, or robust), depression (yes or no), sleep (normal or dysfunctional), urination (normal or dysfunctional), and defecation (normal or dysfunctional). Baseline data were collected from questionnaire interviews, physical examination, clinical records and clinical assessments.

Height and weight were measured in meters and kilograms, respectively, and BMI was calculated by weight divided by the square of height. BMI was categorized using standard BMI categories: underweight (< 18.5 kg/m^2^), normal weight (18.5 to < 24 kg/m^2^), overweight (24 to < 28 kg/m^2^), and obese (≥ 28.0 kg/m^2^) [26]. Previous studies have shown that low grip strength is related to poor cognitive function in the elderly [27]. Low handgrip strength was defined as a handgrip strength of < 26 kg [28]. The Geriatric Depression Scale (GDS) is used to evaluate mental health conditions of the elderly, which includes 15 items. Each item is scored 0 or 1 point. A score of 5 or higher indicates depression, and higher scores indicate greater severity of depression. The scale has been designed specifically for the elderly to help identify those at risk for depression [29].

### Statistical analysis

For the analysis of baseline characteristics, categorical variables included age, gender, ethnicity, marital status, education level, BMI, surgical situation, smoking status, drinking status, bedridden status, falls, handgrip strength, frailty, depression, sleep, urination, and defecation. Variables are described using frequency (percentage), and chi-square tests were used for between-group comparisons. We used a GEE to determine the relationship between cognitive impairment and adverse outcomes, such as death, frailty, and decreased HRQoL, and to control for clustering effects of wards of the same department and confounding effects of demographic characteristics. Odds ratios (OR) and 95% confidence intervals (CI) were calculated to assess the relationship between cognitive impairment and death and frailty. Furthermore, the regression coefficient and 95% CI obtained using Poisson regression of the generalized estimating model were calculated to evaluate the relationship between cognitive impairment and HRQoL. SAS version 9.4 was used for all data analyses with a two-sided significance level of 0.05.

## Results

### Demographic characteristics

**Fig. 1:**
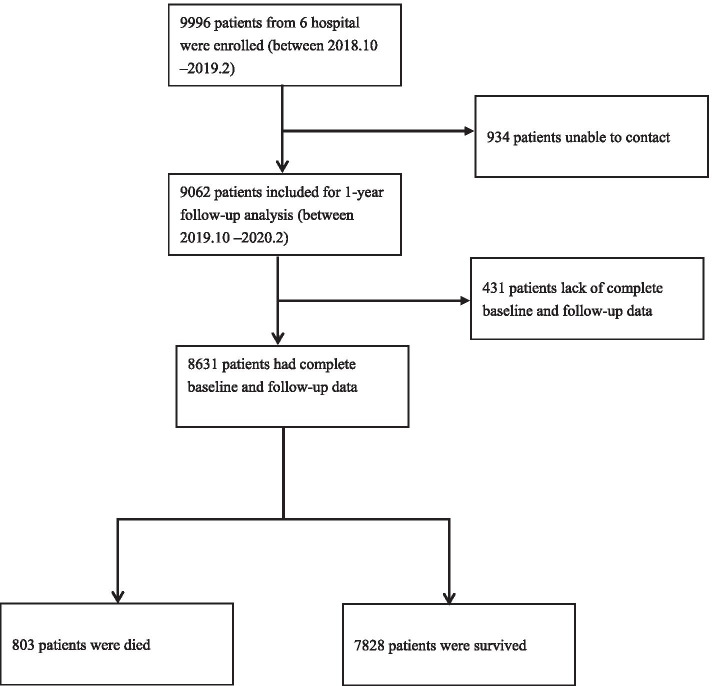
Flowchart of study participants recruitment and follow-up

The flowchart of study participants recruitment and follow-up was shown in Fig. 1. A total of 9996 older patients across six hospitals across China met the study requirements and were included in the study. A total of 934 patients were lost to follow-up and 9062 remained in the cohort after 1-year follow-up. In total, 8631 patients completed the baseline survey and 1-year follow-up. Table 1 shows the baseline information of patients. Of all patients, 70.64% were aged 65-74 years, and 26.27% were aged 75-84 years. There were 5008 men (58.02%) and 3623 women (41.98%), and 94.33% of participants were Han nationality and 88.96% of participants were married. Patients with a middle school degree accounted for 40.46%, and those with a primary school degree accounted for 29.11%. For BMI, 48.6% of patients fell in the normal range, and 34.6% of patients were in the overweight range. One-third of participants had undergone surgery, and 1756 patients (20.35%) had cognitive impairment. According to the FRAIL scale, 39.97% were robust, 43.3% were pre-frail, and 16.73% were frail. Depression was present in 16.09% of patients, low handgrip strength in 51.19%, long-time bedridden in 2.46%, falls in 14.04%, poor sleep status in 42.63%, and urination dysfunction in 13.63%. Patients who smoked and drank alcohol accounted for 11.31 and 11.79% of patients, respectively.

**Table 1:** Prevalence of cognitive impairment across demographic groups (n [%])

	Overall	Cognitive impairment	Cognitive impairment	*P*
Characteristics		No	Yes	
Sample size	8631	6875(79.65)	1756(20.35)	
Age				< 0.001
65–74	6097(70.64)	5020(82.34)	1077 (17.66)	
75–84	2267(26.27)	1681(74.15)	586 (25.85)	
≥ 85	267(3.09)	174(65.17)	93 (34.83)	
BMI				< 0.001
Underweight	575(6.74)	412(71.65)	163(28.35)	
Normal weight	4148(48.60)	3287(79.24)	861(20.76)	
Overweight	2953(34.60)	2437(82.53)	516(17.47)	
Obese	859(10.06)	686(79.86)	173(20.14)	
Gender				< 0.001
female	3623(41. 98)	2711 (74. 83)	912 (25. 17)	
male	5008(58. 02)	4164 (83. 15)	844 (16. 85)	
Ethnicity				
Han	8142(94.33)	6559(80.56)	1583(19.44)	< 0.001
Others	489(5.67)	316(64.62)	173(35.38)	
Education				< 0.001
illiterate	1354(15.69)	801 (59.16)	553 (40.84)	
Primary school	2512 (29.11)	1911 (76.07)	601 (23.93)	
Middle school	3491 (40.46)	3010 (86.22)	481 (13.78)	
University	1272 (14.74)	1151 (90.49)	121 (9.51)	
Frailty				< 0.001
Frail	1444(16.73)	978 (67.73)	466(32.27)	
Pre-frail	3737(43.30)	2946(78.83)	791(21.17)	
Robust	3450(39.97)	2951(85.54)	499(14.46)	
Surgery				< 0.001
No	5828 (67.52)	4535 (77.81)	1293 (22.19)	
Yes	2803 (32.48)	2340 (83.48)	463 (16.52)	
Marital status				< 0.001
Married	7669(88.96)	6203(80.88)	1466(19.12)	
Divorced or widowed	952(11.04)	665(69.85)	287(30.15)	
Smoking status				< 0.001
Current smoker	976(11.31)	797(81.66)	179(18.34)	
Former smoker	1991(23.07)	1635(82.12)	356(17.88)	
Non-smoker	5664(65.62)	4443(78.44)	1221(21.56)	
Drinking				< 0.001
Current drinker	1018(11.79)	860(84.48)	158(15.52)	
Former drinker	1052(12.19)	856(81.37)	1 96(18.63)	
Non-drinker	6561(76.02)	5159(78.63)	1402(21.37)	
Long-time bedridden				< 0.001
No	8419(97.54)	6737(80.02)	1682(19.98)	
Yes	212(2.46)	138(65.09)	74(34.91)	
Falls				< 0.001
No	7419(86. 96)	5970(80.47)	1449(19.53)	
Yes	1212(14.04)	905(74.67)	307(25.33)	
Handgrip strength				< 0.001
low-level	4418(51.19)	3236(73.25)	1182(26.75)	
Normal	4213(48.81)	3639(86.38)	574(13.62)	
Tumor				< 0.001
No	6367(73.77)	4915(77.19)	1452(22.81)	
Yes	2264(26.23)	1960(86.57)	304(13.43)	
Vision				< 0.001
Normal	6845(79.31)	5518(80.61)	1327(19.39)	
Dysfunction	1786(20.69)	1357(75.98)	429(24.02)	
Hearing				0.004
Normal	7089(82.13)	5688(80.24)	1401(19.76)	
Dysfunction	1542(17.87)	1187(76.98)	355(23.02)	
Sleep				< 0.001
Normal	4952(57.37)	4063(82.05)	889(17.95)	
Dysfunction	3679(42.63)	2812(76.43)	867(23.57)	
Urinary function				0.599
Normal	7455(86.37)	5945(79.75)	1510(20.25)	
Dysfunction	1176(13.63)	930(79.08)	246(20.92)	
Depression				< 0.001
No	7172(83.91)	5846(81.51)	1326(18.49)	
Yes	1375(16.09)	960(69.82)	415(30.18)	

### Comparison of older inpatients with and without cognitive impairment

As shown in Table 1, prevalence of cognitive impairment was 20.35%. The prevalence of cognitive impairment was 25.17% for female inpatients and 16.85% for male inpatients respectively. There were significant differences between older patients with cognitive impairment and those with normal cognitive function for age, gender, marital status, ethnicity, education level, BMI, smoking status, drinking status, bedridden status, falls, and sleeping. Prevalence of depression and frailty were also significantly different between the two groups. There was no significant group difference in urination function.

### Association between cognitive impairment and adverse health outcomes

The association between cognitive impairment and 1-year mortality was shown in Table 2. In the unadjusted model, the OR was 1.503 (95% CI: 1.327–1.702) for 1-year mortality in patients with cognitive impairment compared with those with normal cognitive function. After adjusting for age, gender, surgery, frailty, depression, and handgrip strength, compared with patients with normal cognitive function, patients with cognitive impairment had an OR for 1-year mortality of 1.216 (95% CI: 1.076–1.375). The risk of 1-year mortality in participants with cognitive dysfunction was 1.216 times higher than in those with normal cognition, which indicated that cognitive impairment is a risk factor for death.

**Table 2:** GEE analysis of the relationship between cognitive impairment and 1-year death

Variable	Unadjusted			Adjusted*		
	OR	95%CI	*P*	OR	95%CI	*P*
**Total**						
Normal cognition	1(Ref.)	1(Ref.)	–	1(Ref.)	1(Ref.)	–
Cognitive impairment	1.503	1.327–1.702	< 0.001	1.216	1.076–1.375	0.002
**65–74 years old**						
Normal cognition	1(Ref.)	1(Ref.)	–	1(Ref.)	1(Ref.)	–
Cognitive impairment	1.367	1.144–1.633	0.001	1.196	1.001–1.430	0.049
**≥75 years old**						
Normal cognition	1(Ref.)	1(Ref.)	–	1(Ref.)	1(Ref.)	–
Cognitive impairment	1.531	1.261–1.860	< 0.001	1.229	1.009–1.495	0.040

*Adjusted: age, gender, surgery, frailty, depression, and handgrip strength

*GEE* Generalized Estimating Equation

The association between cognitive impairment and 1-year incidence of frailty was shown in Table 3. In the unadjusted model, the OR was 1.476 (95% CI: 1.297–1.679) for 1-year incidence of frailty in patients with cognitive impairment compared with that of patients with normal cognitive function. After adjusting for age, gender, surgery, depression, and handgrip strength, compared with patients with normal cognitive function, patients with cognitive impairment had an OR for 1-year incidence of frailty of 1.195 (95% CI: 1.037–1.376). The risk of 1-year incidence of frailty in older patients with cognitive dysfunction was 1.195 times higher than in those with normal cognition, which demonstrated that cognitive impairment is a risk factor for frailty.

**Table 3:** GEE analysis of the relationship between cognitive impairment and 1-year frailty

Variable	Unadjusted			Adjusted*		
	OR	95%CI	*P*	OR	95%CI	*P*
**Total**						
Normal cognition	1(Ref.)	1(Ref.)	–	1(Ref.)	1(Ref.)	–
Cognitive impairment	1.476	1.297–1.679	< 0.001	1.195	1.037–1.376	0.014
**65–74 years old**						
Normal cognition	1(Ref.)	1(Ref.)	–	1(Ref.)	1(Ref.)	–
Cognitive impairment	1.399	1.172–1.670	< 0.001	1.195	0.989–1.444	0.065
**≥75 years old**						
Normal cognition	1(Ref.)	1(Ref.)	–	1(Ref.)	1(Ref.)	–
Cognitive impairment	1.410	1.152–1.724	< 0.001	1.241	1.015–1.518	0.035

*Adjusted: age, gender, surgery, depression, and handgrip strength

*GEE* Generalized Estimating Equation

The association between cognitive impairment and 1-year HRQoL was shown in Table 4. In the unadjusted model, the regression coefficient for 1-year HRQoL in patients with cognitive impairment was − 0.025 (95% CI: − 0.036−− 0.014) compared with those with normal cognitive function. After adjusting for age, gender, surgery, and frailty, compared with patients with normal cognitive function, the regression coefficient for 1-year HRQoL was − 0.013 (95% CI: − 0.024−− 0.002). Compared with older patients with normal cognition, HRQoL of older patients with cognitive impairment decreased by 0.013 in 1 year. Thus, cognitive impairment was shown to be a risk factor for decline in quality of life.

**Table 4:** GEE analysis of the relationship between cognitive impairment and 1-year HRQoL

Variable	Unadjusted			Adjusted*		
	Coefficient	95%CI	*P*	Coefficient	95%CI	*P*
**Total**						
Normal cognition	0(Ref.)	0(Ref.)	–	0(Ref.)	0(Ref.)	–
Cognitive impairment	−0.025	−0.036 ~ −0.014	< 0.001	−0.013	−0.024 ~ −0.002	0.022
**65–74 years old**						
Normal cognition	0(Ref.)	0(Ref.)	–	0(Ref.)	0(Ref.)	–
Cognitive impairment	−0.019	−0.031 ~ −0.006	0.004	−0.011	−0.023-0.001	0.077
**≥75 years old**						
Normal cognition	0(Ref.)	0(Ref.)	–	0(Ref.)	0(Ref.)	–
Cognitive impairment	− 0.028	− 0.046 ~ − 0.009	0.004	− 0.019	− 0.037 ~ − 0.001	0.045

*Adjusted: age, gender, surgery, and frailty

*GEE* Generalized Estimating Equation

### Stratified association between cognitive impairment and adverse health outcomes by age

As shown in Table 2, risk of death in 1 year is higher in older patients with cognitive impairment who are aged over 75 years compared with those aged 65–74 years. Similarly, as shown in Table 3, older patients with cognitive impairment aged over 75 years have a higher risk of suffering from frailty in 1 year than those aged 65–74 years. Table 4 shows that the decline in HRQoL is greater in the older than the younger age group.

## Discussion

The results of our study showed that there was a significant difference in the incidence of 1-year adverse outcomes (death, frailty, and decreased HRQoL) between older inpatients with cognitive impairment and those with normal cognitive function, which suggested that cognitive impairment is associated with a high risk of adverse health outcomes.

Previous studies have shown that there is considerable overlap between the risk factors of death and cognitive impairment. For example, advanced age is not only an important risk factor for cognitive impairment, but also a risk factor for death in elderly inpatients [31, 32]. This may be because cognitive decline and physical frailty simultaneously promote the occurrence of cognitive impairment and death with age. In addition, results of the study by Georgakis showed a significant increase in all-cause mortality among individuals who had comorbid depression and cognitive impairment compared with those with either cognitive impairment or depression alone, which indicated an interaction between cognitive impairment and depression [33]. Similarly, there was a significant interaction between cognitive impairment and frailty [34]. Because suffering from both physical frailty and cognitive impairment at the same time is common among the elderly, the term ‘cognitive frailty’ has been suggested in recent years to reflect the coexistence of the two diseases [35]. Several studies indicated that there may be a common pathological basis between cognitive impairment and physical frailty, leading to a reduction in life expectancy, such as endocrine dysfunction and systemic inflammation [36]. Some reports found that combining physical frailty and cognitive impairment can improve the possibility of predicting the death risk of the elderly, compared with using either one alone [37]. According to Lee’s study, participants who were frail and cognitively impaired had a 92% greater risk of dying, compared with older inpatients, without frailty and cognitive impairment [38].

Our study showed that after controlling for confounding variables, such as age, gender, depression, frailty, and surgery, the risk of death in older patients with cognitive impairment was 1.216 times higher than in those with normal cognitive function. Adding the above covariates to the model reduced the strength of correlation between cognitive impairment and 1-year mortality. Cognitive impairment can increase the risk of death for several reasons. First, the ability of patients with cognitive impairment to obtain medical information and services and take care of their health, such as adherence to treatment, is limited by their cognitive decline [39]. Second, for the elderly, cognitive impairment is an indicator of general decline in health due to decreased organ reserve capacity, which is associated with increased mortality [40]. Third, cognitive impairment is more common in patients with cardiovascular disease and renal failure. These patients are in poor physical condition, which implies they have a higher risk of death [39].

We found that the risk of suffering from frailty in elderly inpatients with cognitive impairment was 1.195 times higher than those without cognitive impairment [41]. This was consistent with previous studies. As shown in Nyunt’s longitudinal aging study conducted in Singapore [42], the incidence of frailty in the cognitive impairment group (18.5%) was significantly higher than those of the normal low cognition (8.0%) and normal high cognition groups (3.9%). Frailty refers to a biological syndrome in which reserve is diminished and resistance to stress sources is decreased due to the cumulative defect of multiple physiological systems [43, 44]. There is a strong relationship between cognitive impairment and frailty [45]. Cognitive impairment contributes to the occurrence of frailty, which can in turn, lead to cognitive decline [35]. Numerous studies have shown that decreased physical function increases the risk of cognitive decline [46]. When frailty symptoms such as weight loss and slow gait begin to appear, pathological changes such as the amyloid deposition and neurofibrillary tangle formation in the brain and inflammation may leads to the development of cognitive impairment [4, 47].

HRQoL is a multidimensional indicator that is focused on physical and mental health [48]. Recently, HRQoL has been used as an outcome measure in health research because it comprehensively reflects disease conditions and treatment effects, which are important health outcomes for patients. Our study suggested that HRQoL and cognitive function are positively correlated and that cognitive dysfunction increases the risk of HRQoL decline, which is consistent with a previous study that investigated factors related to HRQoL in the elderly [49]. Research has shown that, when cognitive function declines to a certain level, independence of patients with MCI decreases [32].And compared with the elderly with normal cognition, instrumental activities of daily living (IADL) function in MCI patients is poorer [50], which has a significant negative impact on HRQoL [51]. However, our results showed that, the HRQoL of patients with cognitive impairment reduced by 0.013 in 1 year. Although this result was statistically significant, it was not considered clinically significant. A study in Taiwan found that there was no significant difference in HRQoL between cognitive impairment alone and normal cognitive function groups, while the group with frailty has a close correlation with the decline of HRQoL [52]. However, when frailty and cognitive impairment coexist, there may be a multiplier effect on the risk of adverse health outcomes [53]. These two syndromes often occur simultaneously in the elderly. A Japanese study found that among the elderly aged 65 and over living in the community, the prevalence of physical frailty and cognitive impairment was 9.8% [54]. Feng’s research also showed that when participants have both frailty and cognitive impairment at the same time, the risk of decreased HRQoL is much greater than considering these two factors alone [55], indicating that the two affect each other [56].

In addition, the prevalence of mental disease is increasing in the elderly, which should be paid more attention. Depression is an important risk factor for both MCI and decreased HRQoL [57]. However, in the study by Dan Song [48], when depression was added to the regression model, there was no association between cognitive impairment and HRQoL, which may be because depressive symptoms have considerable impact on the cognitive and functional performance of older patients with MCI. Depression may be a mediator between cognitive impairment and HRQoL. The HRQoL of patients with MCI decreased compared with those without MCI. But in comparison, depression plays a more important role in the decline of HRQoL than cognitive ability. The prevalence of depression in patients with cognitive impairment is high, which is reported to be 22.3–63.3% [58]. Depression not only makes people depressed and reduces the quality of psychological life [59], but also accompanied by lack of energy and inattention, which has a great impact on one’s daily life activities, and is not conducive to the disease management of MCI patients [60]. In addition, the elderly with depression tend to report poor self-assessment health [61], which often limits the social participation of the elderly and may indirectly reduce the social and environmental quality of life [62]. Depression is a risk factor for mild cognitive impairment in the elderly [63]. For those with cognitive impairment caused by depression (pseudodementia) [64], antidepressant treatment may restore their cognitive function to normal level.

One strength of our study is the random selection of older inpatients from six provinces or municipalities across China, which provided good representativeness. There have been few large-scale studies on the prevalence of cognitive impairment and its relationship with prognosis in nationally representative older inpatients. Another strength is our use of the generalized estimating model to control for clustering effects of wards of the same department and confounding factors. However, there are also some limitations to the study. We only measured cognitive function at two levels, so we cannot make assumptions regarding the effect of severity of cognitive impairment on risk of adverse outcomes. Previous study classified cognitive function into four groups according to MMSE scores of the subjects: no cognitive impairment, mild cognitive impairment, moderate cognitive impairment, and severe cognitive impairment [65]. Thus, a more detailed classification could be used to explore the impact of severity of cognitive impairment on mortality. Moreover, we used baseline measurement data of cognitive impairment to compare cognitive impairment between the two patient groups, and we did not track changes in severity of cognitive impairment during the follow-up process. It is possible that the relationship between cognitive decline and risk of death may change over time [66, 67]. Additionally, the Mini-Cog is a screening tool rather than a diagnostic tool, and the lack of other diagnostic processes may have overestimated prevalence. Although we adjusted some of the confounding factors, there may be other potential confounders that were not measured at baseline or could not be fully captured [65], which may affect the relationship between prevalence of cognitive impairment and mortality [68]. Lastly, we used all-cause mortality [69] and did not consider the specific causes of death [70]. A clearer relationship between MCI and mortality may be detected by using mortality rates associated with MCI or dementia.

## Conclusions

Our study showed that cognitive impairment is closely related to incidence of 1-year adverse health outcomes (death, frailty, and decreased HRQoL) in older inpatients. Screening the elderly for cognitive impairment at hospitalization is conducive to the development of comprehensive interventions, such as physical exercise [10], which will help prevent the occurrence of adverse outcomes and reduce the burden on patients and caregivers [71].

## Data Availability

The datasets used and analyzed during the current study are available from the corresponding author on reasonable request.

## Abbreviations

MCI: Mild cognitive impairment
GEE: Generalized estimating equation
MMSE: Mini-mental state examination
Mini-Cog: Mini cognitive scale
EQ5D-5L: EuroQol-5 Dimension-5 Level questionnaire
HRQoL: Health-related quality of life
BMI: Body mass index
GDS: Geriatric depression scale
OR: Odds ratios
CI: Confidence intervals
ADL: Activities of daily living
IADL: Instrumental activities of daily living
